# Analytical models for external photon beam radiotherapy out-of-field dose calculation: a scoping review

**DOI:** 10.3389/fonc.2023.1197079

**Published:** 2023-05-09

**Authors:** Nathan Benzazon, Julie Colnot, François de Kermenguy, Samir Achkar, Florent de Vathaire, Eric Deutsch, Charlotte Robert, Ibrahima Diallo

**Affiliations:** ^1^ Unité Mixte de Recherche (UMR) 1030 Radiothérapie Moléculaire et Innovation Thérapeutique, ImmunoRadAI, Université Paris-Saclay, Institut Gustave Roussy, Inserm, Villejuif, France; ^2^ Department of Radiation Oncology, Gustave Roussy, Villejuif, France; ^3^ THERYQ, PMB-Alcen, Peynier, France; ^4^ Unité Mixte de Recherche (UMR) 1018 Centre de Recherche en épidémiologie et Santé des Populations (CESP), Radiation Epidemiology Team, Université Paris-Saclay, Institut Gustave Roussy, Inserm, Villejuif, France

**Keywords:** out-of-field dose, peripheral dose, whole-body dose, analytical models, external radiotherapy

## Abstract

A growing body of scientific evidence indicates that exposure to low dose ionizing radiation (< 2 Gy) is associated with a higher risk of developing radio-induced cancer. Additionally, it has been shown to have significant impacts on both innate and adaptive immune responses. As a result, the evaluation of the low doses inevitably delivered outside the treatment fields (out-of-field dose) in photon radiotherapy is a topic that is regaining interest at a pivotal moment in radiotherapy. In this work, we proposed a scoping review in order to identify evidence of strengths and limitations of available analytical models for out-of-field dose calculation in external photon beam radiotherapy for the purpose of implementation in clinical routine. Papers published between 1988 and 2022 proposing a novel analytical model that estimated at least one component of the out-of-field dose for photon external radiotherapy were included. Models focusing on electrons, protons and Monte-Carlo methods were excluded. The methodological quality and potential limitations of each model were analyzed to assess their generalizability. Twenty-one published papers were selected for analysis, of which 14 proposed multi-compartment models, demonstrating that research efforts are directed towards an increasingly detailed description of the underlying physical phenomena. Our synthesis revealed great inhomogeneities in practices, in particular in the acquisition of experimental data and the standardization of measurements, in the choice of metrics used for the evaluation of model performance and even in the definition of regions considered out-of-the-field, which makes quantitative comparisons impossible. We therefore propose to clarify some key concepts. The analytical methods do not seem to be easily suitable for massive use in clinical routine, due to the inevitable cumbersome nature of their implementation. Currently, there is no consensus on a mathematical formalism that comprehensively describes the out-of-field dose in external photon radiotherapy, partly due to the complex interactions between a large number of influencing factors. Out-of-field dose calculation models based on neural networks could be promising tools to overcome these limitations and thus favor a transfer to the clinic, but the lack of sufficiently large and heterogeneous data sets is the main obstacle.

## 1 Introduction

New technological development in external radiotherapy currently allows, in particular through intensity modulation or stereotactic techniques, an accurate delivery of dose to the target volumes, while minimizing toxic effects on neighboring healthy organs (1, 2). This has led to an increase in overall survival for a large majority of cancer diseases (3). The assessment of the doses delivered outside the high-energy photon treatment field (out-of-field dose) has been an active research area in radiotherapy physics since the 1980s. The increased interest in out-of-field doses coincided with the publication in 1980 of the United Nations Scientific Committee on the Effects of Atomic Radiation (UNSCEAR) report on the Biological Effects of Ionizing Radiations (BEIR) which provided data supporting a slight increase in the risk of radiation-induced second cancers from dose levels of few milligrays [BEIR III (4)]. The implementation of solutions for a comprehensive evaluation of out-of-field doses became therefore essential, initially for epidemiological studies on the potential induction of second cancers at distance from the radiotherapy target volume, in particular among patients treated during childhood (5–8). The concern about the risk of second cancers further increased in recent years with the generalization of intensity modulated radiation therapy (IMRT), significantly increasing the volumes of healthy tissues receiving low doses of ionizing radiation (9, 10), especially since the number of cancer survivors increases (11), in particular among children (12). However, even today, radiotherapy treatment planning systems (TPS) do not allow accurate evaluation of the doses delivered at distance from the target volume because they are specifically commissioned for precise dose calculation within the beams (13). This has been demonstrated for 3-D conformal radiotherapy (13, 14), for IMRT (15–20) and also for the CyberKnife system (Accuray, Sunnyvale, USA) (21, 22).

Different strategies have been developed by radiotherapy physics research teams as an alternative to TPS to precisely estimate out-of-field doses and to provide large amounts of data for epidemiological studies. To this aim, outstanding experimental works have been performed first (23, 24), paving the way to software implementing analytical modeling (25, 26) or implementation of Monte Carlo simulations (27, 28). Yet, to the best of our knowledge, the use of these calculation software remains so far confined to dosimetry for epidemiological research programs. This might be either due to a potential unsatisfactory quality compared to the clinical expectations because of limitations inherent to the implemented models, or simply to the fact that the clinical need is not proven for out-of-field dosimetry.

Regarding the clinical need, the context is now evolving favorably. Indeed, by recommending reporting the dose to the remaining volume at risk (RVR), the International Commission on Radiation Units and Measurements (ICRU) in its report 83 released in 2010, encourages clinicians to widen the dose calculation area so as to encompass the whole patient imaged volume (29). To implement this recommendation, TPS manufacturers will have to upgrade their dose calculation to enable accurate estimation of the dose at greater distances from the target volume than is currently done. In addition, these last years, research on immunotherapy and its association with radiotherapy triggers a renewed interest in out-of-field dose determination, with the goal to assess their potential impact on the patient’s immune system. This is especially important since lymphocytes, including T cells, are known to be particularly radiosensitive (30–33). As a matter of fact, one of the side effects commonly observed in radiotherapy patients is radiation-induced lymphopenia, which is characterized by a significant drop in the quantity of lymphocytes circulating in the blood after irradiation (34). Numerous studies tend to show that severe lymphopenia during radiotherapy is a poor prognostic factor for overall survival and progression-free survival in several tumor locations (35–38). However, the underlying mechanisms related to its severity and duration are still poorly understood, and the effect of irradiation on immune cells in the circulating blood and lymphoid organs, which requires the use of out-of-field dose calculation algorithms, could be an avenue of study for the understanding of radiation-induced lymphopenia, and more generally, the iatrogenic effects of radiotherapy on the immune response (39).

In this work, we conduct, as recommended (40, 41), a scoping review for the purpose of: (1) identifying and defining key concepts, (2) identifying available analytical models for out-of-field dose calculation and evidence of their methodological quality and limitations with regard to their applicability in a clinical workflow, (3) identifying priority areas of work, (4) identifying the role that artificial intelligence and in particular neural networks could play in this context.

## 2 Materials and methods

### 2.1 Key concepts and definitions

**Figure 1:**
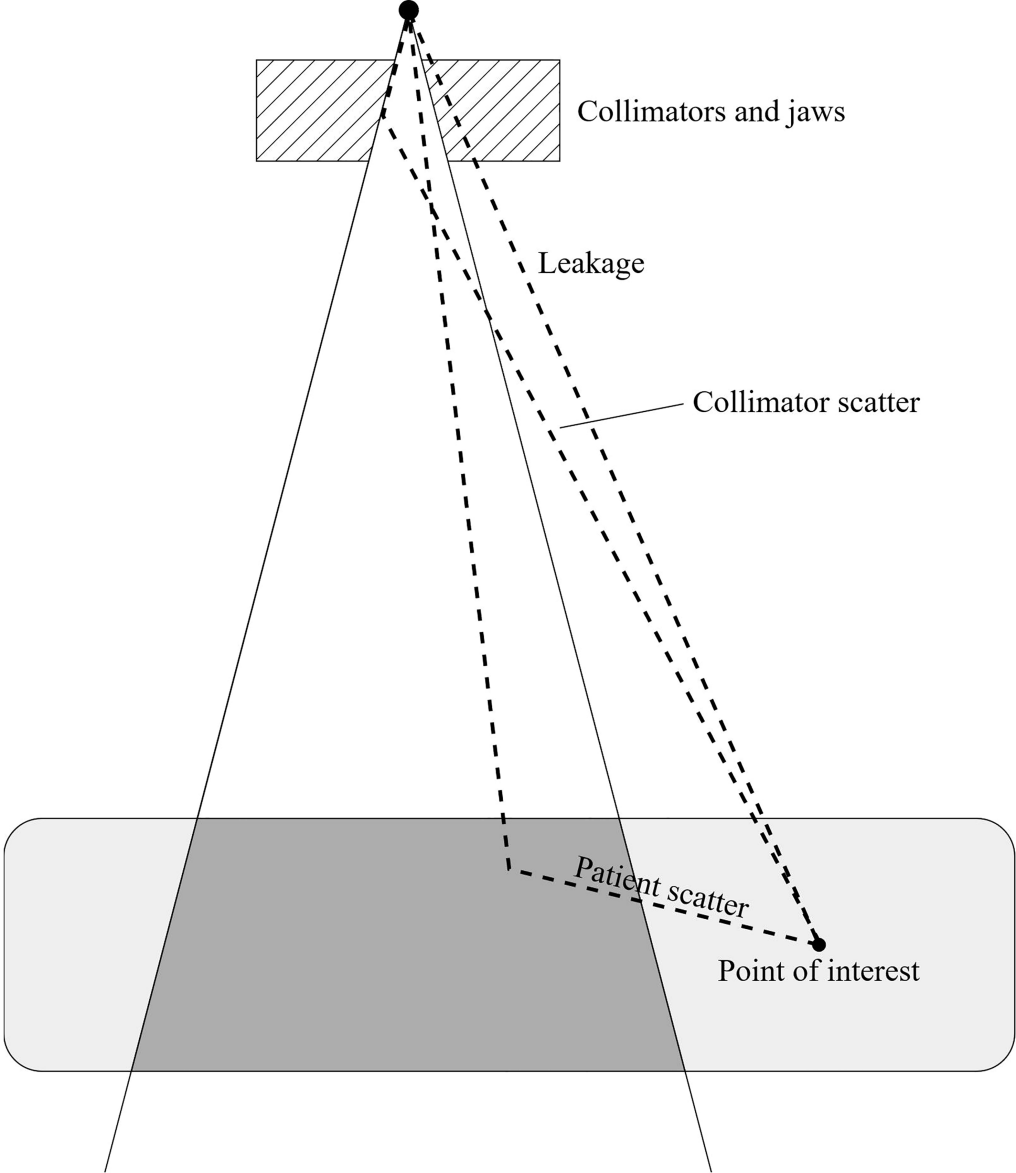
Secondary dose components for a point of interest located outside the treatment field (inspired from (23)).

In 1941, Clarkson (42) proposed a method to calculate the dose at any point within the volume receiving radiation from a photon beam even outside its geometrical limit. The dose was decomposed into two components: the dose due to primary radiation and the dose due to secondary radiation. The doses due to primary and secondary radiation were defined as the dose delivered by a theoretical zero radius circular field, and the additional dose due to the increase in field radius, respectively. Then, in the early 1980s, the group of (23) adapted the notions of primary and secondary radiation for their application on dose outside the photon treatment field. According to Kase, the primary dose is confined within the treatment field while the secondary radiation can be decomposed into three main distinct elements: the patient scatter corresponding to particles which have already interacted once into the treatment field and are scattered; the collimator scatter (or head scatter), corresponding to particles which have already interacted once into the accelerator head and are scattered; and finally, the leakage radiation, which corresponds to primary particles passing through the accelerator head, to finally deposit their energy out of the treatment field. Kase also studied the magnitude of the scatter component from the room and concluded that this component is negligible compared to the three others (**Figure 1**). For medical linear accelerators operating above 10 MV, it is necessary to consider in addition a neutron dose component from (γ, n)-type reactions (43, 44).

### 2.2 Search strategy and inclusion criteria

A search through PubMed/MEDLINE database with the search terms “out-of-field dose”, “stray radiation”, and “peripheral dose” was run. In addition, previous reviews on the topic (45–48) were examined and the list of references of each eligible paper was screened to identify studies potentially missed with the search terms. We included articles published in English, between January 1988 and December 2022, reporting a novel analytical formalism for the evaluation of at least one component of the out-of-field dose in photon external beam radiation therapy (EBRT), or providing information supporting the potential for clinical application of a previously reported model. We have deliberately concentrated our work on photon treatments, as this is the treatment modality still used in the majority of cases today (49). It should be noted that other similar studies have addressed the issue of dose modeling in the scientific literature for other particles (e.g. for protons (50)) and are therefore complementary to this work. Thus, articles focusing on protons or electrons beams were excluded. Similarly, with the goal to move to clinics, we also excluded articles reporting studies primarily designed for out-of-field dose calculations with a MC method and studies limited to experimental investigations. Indeed, if MC simulation is considered by many research teams as the gold standard for dose calculation and has been successfully used in various research areas, the computational resources and time required for dose estimation are too high to be compatible with the constraints of clinical routine, even more so to obtain a sufficiently low statistical uncertainty of type A for voxels located outside the radiation field (51). While significant literature exists on experimental measurements dedicated to out-of-field dose (52), publications that did not perform these for the adaptation of an analytical method were not reviewed.

### 2.3 Data extraction, synthesis, and study quality assessment

The methodological quality and potential limitations of the included articles were assessed with respect to the compatibility of the model with the clinical workflow. We have identified four key criteria that a model must meet in order to be considered compatible with clinical routine workflow. These criteria include accuracy, robustness, calculation time, and convenience. Thus, in addition to accurate dose estimation for each new patient anatomy in a time frame compatible with clinical management, the model must be effective across a wide range of irradiation configurations, while integrating seamlessly into the conventional patient management process, i.e., without imposing an additional burden. A data abstraction form was developed to collect all relevant information: year of publication, country, description of the mathematical formalism of the model, experimental conditions and setup used for measurements, evaluation metrics, associated results and limits. A comments section was used to capture any remaining relevant information not filled in by the existing fields. Missing data were treated as not reported. Data synthesis and study quality assessment were performed independently by two reviewers (NB, ID) who then cross-checked their reports to reach a consensus on quality assessment.

## 3 Results

### 3.1 Selected studies

**Figure 2:**
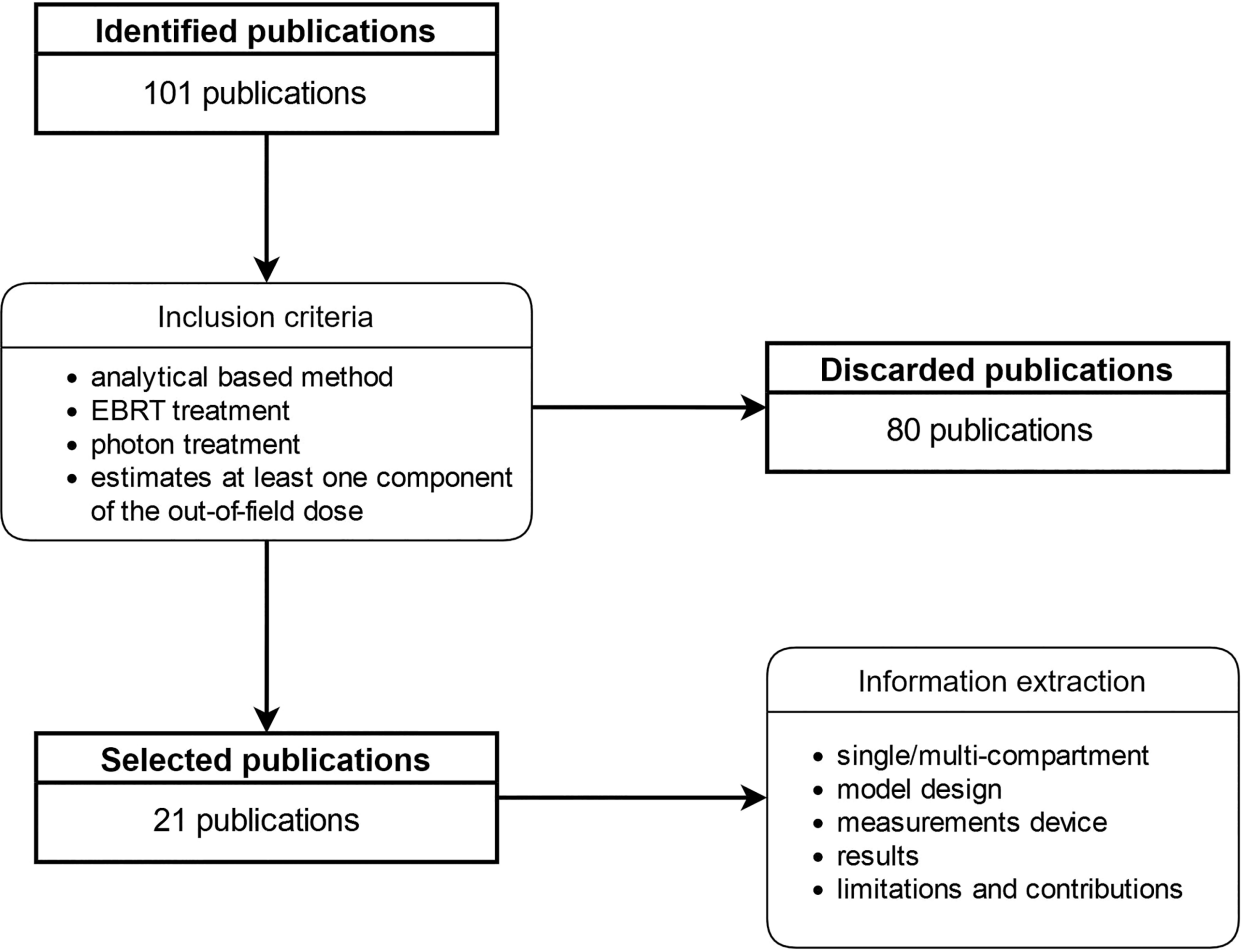
Information extraction and inclusion criteria pipeline.

The flowchart in **Figure 2** depicts the flow of information through our article identification and selection process. 101 publications identified through the database search were reviewed against our inclusion criteria and 21 were retained for synthesis analyses. The median publication year was 2015 (range: 1988-2022) (**Table 1**).

**Table 1:** Methods implemented for acquisitions of experimental data used to set the model basic physical aspects and parameters in the 21 selected articles.

First author name	Year	Experimental conditions		
		Treatment Machine - Manufacturer: [beam energies]	Phantom	Detectors
Francois	1988	Conventional x-rays – Philips [200lVp], Theratron 80 – AECL [^60^Co], Sagittaire – CGR [25 MV]	Water Box/Slabs	0.6 cc IC
McParland	1992	Clinac 6/100 - Varian [6 MV]	Water Box/Slabs	0.6 cc IC
van der Giessen	1993	Theratron-780 – Theratronics [^60^Co]	Water Box/Slabs	0,6 cc graphite IC
van der Giessen	1994	Saturne 41 – GE/CGR [6 MV, 10 MV], Saturne 25 – CGR [10 MV, 23MV], Therac 6 – Theratronics [6 MV]	Water Box/Slabs	0.6 cc nylon IC
Diallo	1996	Theratron 780-AECL [^60^Co], Orion-GE [4MV] and others not specified	In-air	Film
van der Giessen	2001	See van der Giessen 1993 and van der Giessen 1994	N/A	N/A
Benadjaoud	2012	Alcyon II – CGR [^60^Co], Clinac 2300 C/D – Varian [6MV, 20MV], Novalis Tx – Varian [6 MV]	Water Box/Slabs	TLD-700
Taddei	2013	Clinac 2100 – Varian [6 MV], Artiste – Siemens [6 MV]	Anthropomorphic	TLD-100
Veres	2014	See (6)	N/A	N/A
Bezin	2015	Novalis Tx - Varian [6 MV]	In-air	TLD-700
Jagetic	2015	SL25 - Elekta [6 MV], Synergy-S/N:151892 - Elekta	Water Box/Slabs	Diamond detector, IC
Sánchez-Nieto	2015	Primus - Siemens [15 MV, 18 MV], Mevatron - Siemens [23 MV], Clinac - Varian [15 MV], Synergy - Elekta [6MV, 15MV]	N/A	TLD-700, TLD-100
Hauri	2016	Clinac iX - Varian [6 MV], TrueBeam - Varian [6 MV]	Water Box/Slabs	IC
De Saint-Hubert	2017	TrueBeam - Varian [6 MV], Clinac 2100C/D - Varian [6 MV], Hi-Art - Accuray	Anthropomorphic	MCP-N TLD
Schneider	2017	Saturne 43 - GE [6 MV, 12MV, 20 MV], SL25 - Elekta [6 MV, 18 MV, 25 MV], Clinac 21 iX - Varian [6MV], Synergy - Elekta [6MV], Oncor Avant-Garde-Siemens [6MV]	Water Box/Slabs	RPL, OSL, diamond detector, TLD
Yoon	2017	Versa HD - Elekta [6MV FFF, 6MV,10MV FFF, 10 MV], TomoTherapy – Accuray	Anthropomorphic	TLD-100
Gallagher	2018	See (53)	N/A	N/A
Hauri	2019	TrueBeam - Varian [6MV,15 MV]	Anthropomorphic, see (54)	IC and TLD100/100H/700/700H
Schneider	2019	PTS – Elekta [6 MV]	Water Box/Slabs	IC, diode dosimeter
Wilson	2020	See (55)	N/A	N/A
Sánchez-Nieto	2022	N/A	Anthropomorphic	N/A

IC, ionization chamber; TLD, thermoluminescent dosimeter; RPL, radiophotoluminescent dosimeter; OSL, optically stimulated luminescence.

We have referred to Gilbert & Boulter (56) to state our definition of the term *“model”*. Thus, the term *“analytical model”* denotes the mathematical representation, in general simplified, describing the out-of-field dose distribution. In this work, *“Single-Compartment”* refers to models derived by fitting experimental data from in-phantom dose measurements, without addressing each component separately. In contrast, models implementing a mathematical formulation intended to fit the different contributions to the out-of-field dose were categorized as *“Multi-Compartment”*. Both approaches can be based on empirical or semi-empirical/physical mathematical formalisms.

**Table 1** summarizes the methods used for experimental data acquisitions in the 21 selected articles. Seven were reported as single-compartment models: the oldest (25) and the most recent model (57) belong to this category. The remaining articles were multi-compartment models with sub-models dedicated to each of the dose components. One model (43) also included features for secondary neutron dose component.

Although all the models allow the calculation of out-of-field doses for 3-D conformal radiotherapy, only 7 claimed to be suitable for modern techniques as well, in particular IMRT and volumetric arc therapy (VMAT) (**Table 2**). Two articles have a specific approach as they are restricted to model the head scatter component (59) or the head scatter and leakage components (60). Another specific group concerns the articles whose purpose is to implement new features to extend the scope of previously published models, like the utilization of method already developed on new data, the implementation of methods into a software, or into a TPS (61–63).

**Table 2:** Modeling approaches and realistic clinical situations considered in order to test the possibility of clinical transfer of the models presented in the 21 selected articles.

First author	Year	Model typology and out-of-field dose components considered					Clinical examples reported	
		Model compartmentalization	Patient scatter	Head scatter	Leakage	Neutrons	Cancer type or location of the treated tumor	RT techniques
Francois	1988	Single	**✓**	**✓**	**✓**		Pelvic - internal mammary fields	3DCRT
McParland	1992	Multi	**✓**	**✓**	**✓**		N/A	N/A
van der Giessen	1993	Multi	**✓**	**✓**	**✓**		Axilla - mantle field	3DCRT
van der Giessen	1994	Multi	**✓**	**✓**	**✓**		Mantle fields	3DCRT
Diallo	1996	Multi	**✓**	**✓**	**✓**		Breast - Head and Neck	3DCRT
van der Giessen	2001	Multi	**✓**	**✓**	**✓**		N/A	N/A
Benadjaoud	2012	Multi		**✓**	**✓**		N/A	N/A
Taddei	2013	Single	**✓**	**✓**	**✓**		Craniospinal irradiation	3DCRT
Veres	2014	Single	**✓**	**✓**	**✓**		CNS – STS - renal tumor – hepatoblastoma – lymphoma - nasopharynx - testis	3DCRT
Bezin	2015	Multi			**✓**		N/A	N/A
Jagetic	2015	Multi	**✓**	**✓**	**✓**		N/A	N/A
Sánchez-Nieto	2015	Multi	**✓**	**✓**	**✓**		Head and neck – prostate	IMRT-VMAT
Hauri	2016	Multi	**✓**	**✓**	**✓**		Prostate	3DCRT-IMRT-VMAT-FFF
De Saint-Hubert	2017	Single	**✓**	**✓**	**✓**		Craniospinal irradiations	3DCRT-IMRT-HT
Schneider	2017	Multi	**✓**	**✓**	**✓**		Prostate, see (58)	3DCRT-IMRT-HT-STEREO
Yoon	2017	Single	**✓**	**✓**	**✓**		Post-mastectomy radiotherapy	IMRT-VMAT-FFF
Gallagher	2018	Single	**✓**	**✓**	**✓**		Brain	3DCRT
Hauri	2019	Multi	**✓**	**✓**	**✓**	**✓**	Prostate.	3DCRT-IMRT-VMAT-FFF
Schneider	2019	Multi	**✓**	**✓**	**✓**		N/A	N/A
Wilson	2020	Multi	**✓**	**✓**	**✓**		Prostate	3DCRT
Sánchez-Nieto	2022	Single	**✓**	**✓**	**✓**		Prostate – lung	3DCRT - VMAT

CNS, central nervous system; STS, soft tissue sarcoma; FFF, flattening filter free; 3DCRT, three-dimensional conformal radiation therapy; IMRT, intensity-modulated radiotherapy; VMAT, volumetric modulated arc therapy; HT, helical tomotherapy; STEREO, stereotactic radiation therapy.

### 3.2 Experimental device

The characteristics of the treatment machines investigated, the phantoms and the detectors used are summarized in **Table 1**. Among the 21 selected articles, 16 detailed the experimental device used for the acquisition of input data for the model development. The remaining 5 are about implementation of already existing models.

#### 3.2.1 Treatment machines investigated

Depending on the research groups, measurements were performed by an orthovoltage unit operated at 200 kVp and a cobalt-60 machines (25), different medical accelerators operated at 6 MV (20, 53, 57, 61, 64), 10 MV (20) and 25 MV (25). Measurements could have been made for a set of simple geometry open square and rectangular fields (25, 65) or, for a set of reference treatment plans-specific beam settings (20, 53, 57, 61, 64).

#### 3.2.2 Phantoms

The analytical models presented all share the need for absorbed dose measurements at a set of carefully selected relevant out-of-field locations either in a simple geometry water or water-equivalent solid phantom or in an anthropomorphic phantom. In addition, in-air measurements are sometimes carried-out by some authors (6, 59, 66, 67). Among the experimental methods reported, the tendencies in decreasing order of frequency, appeared to be the use of a water tank or a water equivalent slab phantom of simple geometry (9 out of 16 publications), an anthropomorphic phantom (5 out of 16) or even direct in-air measurements (2 out of 16).

#### 3.2.3 Detectors

Ionization chambers and thermoluminescent dosimeters (TLDs) are the two most commonly used types of detectors for these measurements, with similar frequencies of use, each being involved in 50% of the publications. In case of measurements in an anthropomorphic phantom, TLDs of type TLD-100 (20, 53, 57, 61) or an ultra-sensitive MCP-N type TLDs (64) were employed. Diamond detectors (55, 63, 67) or solid-state diode dosimeter (67) were also used. Less often, radiophotoluminescent (RPL) and optically stimulated luminescence (OSL) detectors were involved (55). In specific situations where the objective was to assess secondary neutrons contribution to the out-of-field dose, combinations of different TLDs types i.e. TLD100 (LiF:Mg,Ti), TLD100H (LiF:Mg,Cu,P), TLD600/700 (LiF:Mg,Ti) and TLD700H (7LiF:Mg,Cu,P) were used synergistically (43, 68). In addition, MC simulations were performed by some groups, as a surrogate or to supplement their experimental data (43, 57, 67).

### 3.3 Single-compartment models

*Experimental protocol to measure all the components of the out-of-field dose as a whole.*

The methodologies implemented by the different research groups for single-compartment models development consisted in an experimental measurement campaign of the total out-of-field dose.

#### 3.3.1 Mathematical formalisms

Two types of models can be observed:

One approach discriminates the near field and the distant regions (25, 53, 57, 61, 64). In this case, the sector integration method, proposed by (42) and investigated by (69), was implemented by the Gustave Roussy’s group (25), whilst the group from MD Anderson Cancer Center (53, 61, 64) opted for the double-Gaussian description originally proposed by (70). The group from the Pontificia Universidad Católica de Chile (57) used an expression combining an exponential attenuation and the inverse square law. For distant regions, formula fitting the isodoses using either a combination of parametric elliptical equations (25), a (70) Gaussian function (53, 61, 64), or a constant background (57) were implemented. Let us illustrate this model family with (53). In (53), the normalized out-of-field dose D (in cGy.Gy^-1^) was expressed as a function of the distance from the field edge ( r ), by the sum of two gaussians (equation 1). The resulting absorbed dose, $D_{model}$ , correspond to the absorbed dose normalized to the prescribed therapeutic dose.


(1) $$D_{model}=\frac{\alpha_{1}}{\sqrt{2\pi \sigma_{1}^{2}}}e^{\frac{{\left(r-{\text{μ}}_{1}\right)}^{2}}{2\sigma_{1}^{2}}}+\frac{\alpha_{2}}{\sqrt{2\pi \sigma_{2}^{2}}}e^{\frac{{\left(r-{\text{μ}}_{2}\right)}^{2}}{2\sigma_{2}^{2}}}$$

With ${\text{µ}}_{1}$ and ${\text{µ}}_{2}$ the means of each gaussian, $\sigma_{1}$ and $\sigma_{2}$ the associated standard deviations, and $\alpha_{1}$ and $\alpha_{2}$ two magnitude factors. In this expression, one gaussian modeled the dose near the field edges, and the second one modeled the dose at a greater distance from the field.

Another one addressed the whole volume by a unique formula (20, 65). The out-of-field dose was thus deduced from the central axis depth dose or by the prescribed dose by applying a multiplicative coefficient defined as a polynomial function of order two depending on the inverse of the distance to the edge of the field (20) or from out-of-field dose profiles obtained from water phantom measurements (65). Model developed by (20) illustrates this approach. In (20), the out-of-field dose was mathematically expressed as follows:


(2) $$\text{D}={\text{D}}_{RX}\times \left[\frac{{\text{C}}_{1}}{(\text{d}+\text{t}{)}^{2}}-\frac{{\text{C}}_{2}}{\left(\text{d}+\text{t}\right)}+{\text{C}}_{3}\right]$$

With ${\text{D}}_{RX}$ the prescription dose, d the distance from the field edge, and ${\text{C}}_{1}$ , ${\text{C}}_{2}$ , ${\text{C}}_{3}$ and t fitting parameters.

### 3.4 Multi-compartment models

*Experimental protocol implemented to separate the o*ut-of*-field dose components*

**Figure 3:**
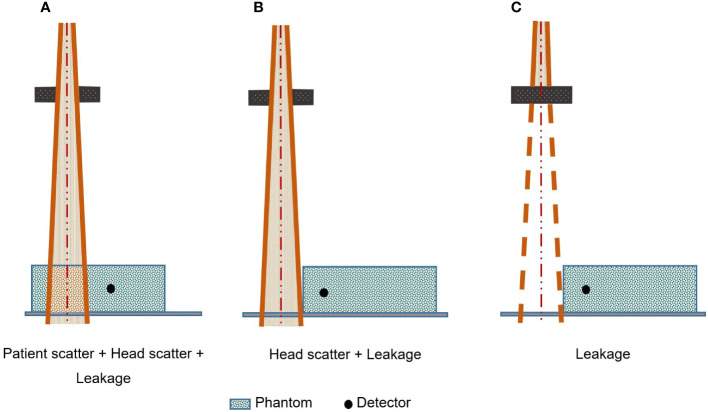
Kase’s experimental protocol in order to estimate different out-of-field-dose components separately [inspired from (23)].

All the research groups proposing multi-compartmental models, except (6) and (59), have referred to the (23) experimental protocol. In this protocol, illustrated in **Figure 3**, collimator scatter and head leakage are measured in water by directing the primary beam outside the phantom, the head leakage being assessed by closing the jaws. The contribution of patient scattering is evaluated by subtracting the measurements performed with the primary beam irradiating the phantom from the values obtained previously with the open field. Other strategies were proposed such as in-air measurements to map the incident photons energy fluence around the treatment beam (6), to assess collimator scatter and head leakage photons characteristics (26) or to map head leakage (59).

#### 3.4.1 General characteristics and model design

Semi-empirical models for out-of-field dose calculation are typically designed in the form of a two-stage mathematical formalism: an empirical description of the photon fluence based on a simplified representation of the treatment head is combined with a simplified physics theory regarding radiation and matter interaction to derive the absorbed dose distribution in the patient. In a more empirical approach, the Gustave Roussy’s group model (6) escapes any representation of the treatment head by choosing direct measurements of attenuation curves around the treatment beam with films to derive an extended empirical model of the spatial distribution of the incident photons fluence. This leads to account for head leakage and scatter, and even scatter from the treatment room as well, as a whole.

#### 3.4.2 Describing head scatter

The spatial distribution of the photon fluence resulting from the treatment head scatter was modeled by assimilating either the treatment machine head with a virtual isotropic radiation point source (68), or with a combination of virtual plan sources (26, 60). In these two last papers, angular distribution of scattered photons was driven by the Henyey-Greenstein phase function (71) and photon energies followed the standard Compton scattering expressed by the Klein–Nishina formula (72, 73). Like in other models (66, 67), it was assumed that the primary photons were emanating from a virtual source located near the target with a spatial fluence distribution driven by the sum of several Gaussians. In contrast, an empirical formula was developed by the University of Zurich group (26) assuming a field size-dependent virtual source for the collimator scattering located at the rear jaw. Alternatively, a group from Louisiana State University and Agricultural and Mechanical College (55) reported another descriptive analytical formula accounting for the dependence of the head scatter upon beam energy, depth, and field size, through a combination of linear and exponential functions.

#### 3.4.3 Describing leakage

The most sophisticated mathematical formalisms to derive the leakage component (66, 67) address separately the transmission through the primary collimator, the flattening filter, the secondary collimator, the jaws and the MLC, taking into account the geometrical and physical characteristics of these elements, through an exponential decrease. In an extensive study of the leakage component, a model used a graphics library and 3D rendering to model the linear accelerator shielding elements and accelerating waveguide, and implementing the build-up factor corrected by the exponential attenuation (59). However, most of the leakage models were greatly simplified as it has been modeled either by a constant background dose (68) or as originating from a field size independent virtual source at the target location (26, 60). An alternative approach considered that the leakage was attenuated by the secondary collimator close to the primary beam and additionally by the primary collimator in distant regions (55).

For instance, reference (68) modeled the head scatter and leakage components as a whole as follows:


(3) $$PPD\left(x,z,f,\varepsilon \right)=A+\frac{B}{x^{2}}\varepsilon F\left(f\right)e^{-\left({\text{µ}}_{air}.r_{air}+{\text{µ}}_{tissue}.r_{tissue}\right)}$$

With PPD the peripheral dose per machine unit (MU) due to leakage and head scattered photons, that depends on the distance to the treatment isocenter ( x ), depth ( z ), field size (f), and ϵ, which is a correction factor for treatment efficiency that takes into account the differences of MU when using a modulated treatment compared to a reference treatment for the same delivered dose. F(f) represents a correction factor for field sizes. A represents the leakage component, and B  is an adjustment parameter. ${\text{µ}}_{air}$ and ${\text{µ}}_{tissue}$ are respectively the linear attenuation coefficients in air and tissues, while $r_{tissue}$ and $r_{air}$ being respectively the distance travelled in tissue or air.

#### 3.4.4 Describing patient scatter

To model patient scatter, diverse options were considered: formula combining the Klein-Nishina coefficients for Compton scattering, the inverse square law and attenuation factors (74) and annihilation photons (75). Some authors assumed a virtual circular source of photons located on the beam central axis and consisting of infinitely small sources. The contributions from each infinitely small source are attenuated exponentially and the inverse square law is applied (26). Similarly, a virtual point source combined with transmission factors in water (66) or a single exponential component (76) were assumed to describe patient scatter. Another alternative was an empirical expression implementing different beam energy- and depth-dependent scaling factors to adjust the transmission and attenuation for radiation from patient scattering (55). Some authors have even chosen to neglect the patient scatter dose and have therefore limited the range of validity of their model to the volumes beyond 10 cm from the field edge (68), which present important limitations, especially for treatments with large fields, where patient scatter can have a major contribution well beyond 10 cm (23, 77). Let us illustrate this representation with (74) which analytically modeled the patient scatter component of the out-of-field dose as follows:


(4) $${\text{D}}_{\text{psc}}\left(\text{Q}\right)={\text{C}}_{\text{KN}}\times {\text{C}}_{\text{d}}\times {\text{C}}_{\text{att}}\times \text{Φ}\left(\text{P}\right)\times {\text{E}}_{\text{sc}}\times {\text{μ}}_{\text{en}}$$

With ${\text{D}}_{\text{psc}}\left(\text{Q}\right)$ the patient scattering component from P at point Q , ${\text{C}}_{\text{KN}}$ the Klein-Nishina coefficient, ${\text{C}}_{\text{d}}$ the inverse square law corresponding to the distance d , ${\text{C}}_{\text{att}}$ the attenuation coefficient, Φ(P) the photon fluence at point P , ${\text{E}}_{\text{sc}}$ the energy of scattered photons and ${\text{μ}}_{\text{en}}$ the mass energy absorption coefficient.

#### 3.4.5 Describing secondary neutrons contribution

Authors studying out-of-field doses for beam energies greater than 10 MV systematically mention the existence of photoneutrons but consider that the equivalent dose from photoneutrons is negligible compared to that generated by photons. Particular attention should however be given to the results of the University of Seville group (78) which reported neutron equivalent doses obtained on 1377 adult patients in more than 30 different institutions using a digital neutron detector located inside the treatment room (79). The research team pointed out that although the neutron dose contribution inside the treatment field was negligible, it contributed to the total dose outside the field by about 25%, with this value decreasing rapidly with depth. Beyond 50 cm from the treatment field, the neutron dose is of the same order of magnitude as the leakage (43). Thus, neutron contribution should not be neglected, especially far away from the treatment field (43, 80, 81). The amount of neutrons produced has been shown to correlate with the complexity of the linac head, e.g., the presence of jaws and MLC (80), and with the number of MU (81).

However, we identified models in which neutrons were not taken into account in spite of energies used greater than or equal to 10 MV (25, 55) and only one paper provided an analytical model for the neutron component. In this aim, the University of Zurich (43) assumed a point source of neutrons located in the target and converted the fluence to a neutron equivalent dose through the Siebert and Schumacher quality factors (82).

### 3.5 Model performance evaluation and inter-comparability

Our investigation revealed that several factors make quantitative comparisons of the performance of the reported analytical models impossible.

First, the definition of out-of-field dose depends on the authors. The following definitions were reported:

- Doses below 5% of the prescribed dose (13, 19, 57, 67, 83),
- Doses below 50% of the prescribed dose (20, 53, 64).
- An alternative approach consisted in excluding all voxels where the TPS calculated dose was above 50% of the prescribed dose, and within the beam path and considering all remaining locations as out-of-field (63).
- In addition, some authors (43) defined the measurement points locations for model development or evaluation according to specific recommendations (84).

Our investigation also revealed that dose reporting rules depended on the research group, with measurements normalized in different ways. For instance, doses relative to the maximum central axis dose (60), or relative to the prescribed dose (53) have been reported depending on the studies.

Finally, model performances were assessed and reported in many ways, i.e., using:

- the root mean square deviation (RMSD) (20, 53, 61, 64, 66, 68),
- the mean, maximum or median differences both absolute and relative (6, 25, 26, 43, 55, 60, 66, 74, 75, 85),
- the mean ratio (62, 86)
- a generalized gamma index including an additional parameter for the low dose regions (87) was preferred in several studies (55, 63, 66, 67).

This observation makes it very difficult to compare the performance of the models, especially as this performance is quantified by comparing predictions with measurements, for which the number of experimental points and the position of the detectors vary drastically from one study to another.

Our investigation showed that TLDs were the most widely used dosimeters for assessing model performance through anthropomorphic phantom measurements (**Table 1**) in spite of their limitations. For instance,TLD-100s associated with high voltages superior to 15 MV (88), can overrespond by 4% to 12%, and correction factors are required (89).

### 3.6 Model versatility

A series of limitations related to model versatility have been identified by the authors themselves. Among these, extensive out-of-field dose measurements required for model implementation are technically demanding and laborious. Inclusion of neutron contribution is possible but nontrivial and at the cost of much more demanding measurements (76). Nevertheless, an out-of-field dose calculation model initially developed for static radiotherapy and IMRT with a 6 MV photon beam (26) has been successfully improved and adapted to incorporate a neutron equivalent dose component through additional measurements at 15 MV (43). A broadly applicable method was developed by generalizing an existing physics-based method, in which the model may be adapted for a large variety of devices without using proprietary configuration parameters, but with easily accessible dosimetric data (55). It appears to be a strong argument showing the relevance of future research towards a universally applicable model for estimating the out-of-field dose. Our analysis, however, evidenced that, the more a model is fitted to heterogeneous data, i.e., from different accelerators, or corresponding to different energies or irradiation techniques, the worse the performance will be. For instance, the generalization of a model initially developed from data obtained with a ^60^Co machine (74) to irradiation configurations from 6 MV, 10 MV and 23 MV linear accelerators (75) has resulted to a 20% loss of accuracy.

### 3.7 Clinical translation ability

As shown in **Table 2**, we found as expected that the further we go in time, the more frequent are the authors who claimed that their model would be suitable for complex radiotherapy techniques, in particular IMRT and VMAT. A total of 8 models are reported to be compatible with modern radiotherapy techniques (20, 26, 43, 55, 57, 60, 64, 68). Among these, clinical examples were reported for all but (60), because this last was restricted to the modeling of the MLC system. We can also notice that devices such as CyberKnife, Gamma Knife (Elekta Instruments, Stockholm, Sweden), TomoTherapy unit, or Halcyon (Varian Medical Systems, Palo Alto, USA) have been little used for the realization of measurements in the context of the development of analytical models (see **Table 2**). Nevertheless, it should be noted that some publications have addressed the assessment of out-of-field dose from an experimental point of view for the Halcyon device (15), for the Gamma Knife (90), or for CyberKnife and TomoTherapy devices (22). Several groups have developed bridging techniques with hybrid approaches using TPS-estimated doses for the in-field dose and analytical models for the out-of-field dose to obtain a whole body dose estimate (26, 43, 53, 61, 63, 65). Regarding the computation time, which is a critical parameter for clinical transfer, a few authors presented clear values. Reported computation times were 8 hours and 10 minutes per patient on a single CPU or a 64-core workstation (61), and four minutes (66) or 9 minutes per field (26).

## 4 Discussion

The aim of this exploratory research was to examine, through the available scientific publications, the key concepts underlying the analytical models dedicated to out-of-field dose calculation in external photon radiotherapy and to summarize the current state of the art in this field. Our work provides an overview of the typology of existing models, the extent of available experimental data and the knowledge to date.

First, existing analytical models for out-of-field dose calculations fall into two categories. Single-compartment models include all out-of-field dose components into a single general empirical mathematical formula. In contrast, multi-compartment models separate patient scatter from collimator scatter and leakage. Fourteen (almost 67%) out of the 21 selected articles reported multi-compartment models, showing that research efforts are directed towards an increasingly detailed description of the underlying physical phenomena. We have identified remarkable efforts to model the out-of-field dose component related to the photons scattering on the treatment beam collimating system (60), transmission and leakage through the beam limiting device (67) and also scattering in the patient (26).

Unfortunately, there is a lack of homogeneity in the definitions of some basic concepts, in particular the definition of the out-of-field dose itself. This, together with the lack of homogeneity in the normalization processes and the metrics used to present the results, makes it impossible to quantitatively compare model performance. It is therefore difficult to identify methods that are more efficient than others. Nevertheless, the synthesis that we provided in the paragraph devoted to general concepts and definitions are a step in encouraging the different research teams involved in this research field to move towards common practices. The practices that stand out the most could be accepted as the standard approach - such as the definition of the out-of-field as the dose below the 5% isodose, or the RMSD as the conventional performance evaluation metric. Of note, the recommendation for evaluating the dose to the RVR (29) is also an important step towards the harmonization of the practices. In addition, we note that the uncertainties related to the measurement processes allowing the fit of the analytical methods were not described in the analyzed publications, which makes it difficult to assess their accuracy. In (91), it was clearly stated however that one of the main sources of uncertainty in the estimation of the out-of-field dose comes from the measurements themselves. Indeed, these uncertainties are known to be higher than in-field ones, due to the unknown photon spectra. The maximum overall uncertainty in out-of-field dose measurements has been reported to be about 15% by authors using TLDs, ionization chambers, and radiophotoluminescent dosimeters (92). The creation of an international working group to provide guidelines regarding good practices would allow an harmonization of reported results, necessary for a more widespread use of this research topic in clinical practice. Let us note the Working Group 9 (WG9 - Radiation dosimetry in radiotherapy) of the European Radiation Dosimetry Group, which is already interested in out-of-field doses in brachytherapy.

Finally, the problems of versatility of most of the analytical methods, and the cumbersome nature of their implementation, is undoubtedly an obstacle to their widespread use in clinical routine, despite their obvious clinical interest and the fact that UNSCEAR emphasized the need for a systematic assessment of out-of-field doses in relation to second primary cancer after radiotherapy during its 69th session in 2022. The new possibilities offered by artificial intelligence methods, that are already revolutionizing various fields in radiotherapy planning (93–95), could be an interesting avenue to overcome this drawback. Neural Network-based models (NNs) have proven good performance in solving complex tasks, for various applications in medical physics, such as automatic segmentation (96), automatic planning (97) or the generation of virtual images (98), but, to our knowledge, there is currently no NNs developed for out-of-field dose calculation, although they have already proven their ability in estimating the in-field dose (95, 99–103). It should be noted, however, that the current lack of a publicly available dataset large enough and versatile enough to properly train a neural network may be a serious obstacle that will be difficult to overcome.

While there have been a number of interesting reviews published on similar topics in the past, it’s worth noting that our paper takes a unique approach and offers a fresh perspective. For instance, the work of (48) gave an almost exhaustive overview of the applications of the available methods for dose reconstructions for therapeutic and diagnostic exposures for use in retrospective epidemiological studies. This publication has of course examined all the outstanding analytical models used before 2006 for dosimetry in retrospective epidemiological studies (47). provided a slightly more recent overview on dosimetry for second cancers studies, while (46) analyzed the main contributing factors to out-of-field doses, including linear accelerator type, field size, beam quality, leakage, collimator scatter, treatment technique, and their clinical implications, with the aim of highlighting ways to reduce the risk of associated radiation-induced cancers. Even more recently, a review presenting existing data from measurements and calculations of out-of-field doses in external photon beam radiotherapy and discussing its clinical implications has been published (45). Our research stands out as original due to its scoping review design. This approach complements and enriches the existing scientific literature by providing up-to-date, and specific information on all published mathematical models. This information is crucial for designing future research that aims to develop a universally applicable software package for out-of-field dose calculations usable in clinical practice.

## 5 Conclusion

In this scoping review, we have comprehensively presented the published articles on analytical models for out-of-field dose calculation in photon external radiotherapy and highlighted the remarkable efforts that have been made in the field. Through this work, we have also highlighted some of the pitfalls that currently hinder their widespread use in clinical workflow and the need for standardization in order to simplify comparisons. In a clinical context where modulated irradiation is becoming the standard of care and where combination treatments suggest that doses received by healthy organs located at a distance from the irradiation field may play a key role, efforts must be made to find solutions versatile enough to be compatible with clinical practice. The neural network approach could be a relevant candidate provided that an adequate data set is available.

## Author contributions

NB collected the data, analyzed publications, drafted the figures and table, and wrote the manuscript. SA and FdK gave targeted expertise. CR, ID, and JC helped with thematic expertise and for the publication structure. CR and ID helped with the general writing and the structure of the document and gave final approval of the submitted version. All authors contributed to the article and approved the submitted version.
